# Correlation between serum autotaxin levels and optic neuropathy in early diabetic retinopathy: a case-control study

**DOI:** 10.3389/fmed.2025.1553136

**Published:** 2025-05-30

**Authors:** Wenjun Chen, Xiaori Chen, Yaming Wang, Chuanheng Guo, Shaowen Yu, Wen Zhao

**Affiliations:** ^1^Department of Ophthalmology, Qingdao West Coast New District Central Hospital, Qingdao, China; ^2^Department of Physiatry, Qingdao West Coast New District Central Hospital, Qingdao, China; ^3^Department of Ophthalmology, Traditional Chinese Medical Hospital of Huangdao District, Qingdao, China

**Keywords:** serum autotaxin levels, early stage, diabetic retinopathy, optic neuropathy, correlation

## Abstract

**Objective:**

To explore the correlation between serum autotaxin (ATX) levels and optic neuropathy in early diabetic retinopathy (DR).

**Methods:**

A total of 90 patients with early DR who were treated in our hospital from August 2022 to December 2023 were selected as the Non diabetic neuropathy (NDN) group, and another 90 patients with early DR combined with optic neuropathy were selected as the combined diabetic neuropathy (DN) group. In addition 90 healthy patients were selected as a normal control group. The general data of the two groups of patients were collected, and the levels of inflammatory factors were compared, including the platelet-to-lymphocyte ratio (PLR), neutrophil-to-lymphocyte ratio (NLR), lymphocyte-to-monocyte ratio (LMR), systemic inflammation response index (SIRI), platelet-to-albumin ratio (PAR), serum C-reactive protein (CRP), tumor necrosis factor-α (TNF-α), and interleukin-6 (IL-6). The levels of ATX, glial fibrillary acid protein (GFAP), and neurofilament light chain protein (NfL) were also compared between the two groups. Retinal OCT examination was performed in both groups to record the foveal avascular zone (FAZ) area, acircularity index (AI), mean RNFL thickness at the disk margin, temporal, superior, nasal, and inferior, and mean macular ganglion cell inner plexiform layer (mGCIPL) thickness and vascular density (VD) levels. Pearson analysis was used to analyze the correlation between ATX and inflammatory factors, GFAP, NfL, mGCIPL, and VD levels. Multivariate Logistic regression analysis was used to analyze the influencing factors of optic neuropathy in DR patients. ROC curve analysis was used to analyze the clinical value of ATX in diagnosing optic neuropathy in DR patients.

**Results:**

There were no significant differences in PLR, NLR, LMR, PAR, SIRI index, serum TNF-α, CRP, and IL-6 levels between the healthy group, the NDN group and the combined DN group (*P >* 0.05). However, serum ATX, GFAP, and NfL levels in the combined DN group were significantly higher than those in the healthy group and the NDN group, and the difference was statistically significant (*P <* 0.05). The mGCIPL and VD levels of the combined DN group were significantly lower than those of the healthy group and the NDN group, and the difference was statistically significant (*P* < 0.001). Pearson correlation analysis showed that the ATX level of DR patients was positively correlated with GFAP and NfL (*P* < 0.05), and negatively correlated with mGCIPL and VD levels (*P* < 0.001). The results of multivariate logistic regression analysis showed that serum ATX, GFAP, and NfL levels were independent risk factors for optic neuropathy in DR patients (*P* < 0.05), and GFAP and NfL were independent protective factors (*P* = 0.001). The results of ROC curve analysis showed that the area under the curve (AUC) of ATX for diagnosing optic neuropathy in DR patients was 0.873, with a 95% CI of 0.808–0.938. When the ATX cut-off value was 4.69 ng/mL, the maximum Youden index was 0.635, with a sensitivity of 71.11% and a specificity of 93.33%. It has certain clinical value in diagnosing optic neuropathy in DR patients.

**Conclusion:**

The increase of serum ATX content may be involved in the occurrence and development of optic neuropathy in DR, and has a significant correlation with early neurological and vascular changes in the early stage of the disease. Measuring serum ATX levels may aid in the early diagnosis of optic neuropathy in DR and provides new ideas for its treatment.

## 1 Introduction

Diabetic retinopathy (DR) is one of the most common microvascular complications of diabetes and the most significant manifestation of diabetic microangiopathy. It is a series of fundus lesions with specific changes caused by retinal microvascular leakage and blockage due to chronic progressive diabetes (1, 2). Another study showed that the estimated prevalence of diabetic retinopathy in hospitalized patients was 44%, and the prevalence of previously undiagnosed diabetic retinopathy and vision-threatening retinopathy was 25 and 19%, respectively (3). Depending on whether retinal neovascularization occurs, diabetic retinopathy is clinically divided into non-proliferative diabetic retinopathy (NPDR) and proliferative diabetic retinopathy (PDR). NPDR refers to diabetic retinopathy without retinal neovascularization, while PDR refers to diabetic retinopathy with retinal neovascularization (4). In the past, DR was believed to be mainly a vascular lesion, but with the deepening of research, it was found that neural damage occurs in the early stages of DR and is closely related to function (5). Current studies indicate that DR is a multi-organ disease influenced by the interaction between neural and vascular components, where neurons, glial cells, and the vascular system form an interdependent entity known as the retinal neurovascular unit (NVU) (6). Therefore, the detection and evaluation of the degree of retinal neurodegeneration and the clarification of the relationship between neurodegeneration and microvascular damage has become the focus of current research on DR. Circulating biomarkers play an important role in the diagnosis and treatment of DR. At present, some circulating biomarkers that may be related to DR have been found. Serum autotaxin (ATX) is a secretory glycoprotein with phospholipase D activity that can catalyze the hydrolysis of lysophosphatidylcholine (LPC) to generate lysophosphatidic acid (LPA) (7). LPA is a lipid mediator with multiple biological activities that participates in cell proliferation, differentiation, migration, and inflammatory response. Recent studies have shown that ATX and LPA play an important role in the occurrence and development of many diseases, including diabetes and its complications (8). However, there are currently few studies on the correlation between serum ATX levels and optic neuropathy in early DR. Based on this, this study aimed to explore the correlation between serum ATX levels and optic neuropathy in early diabetic retinopathy.

## 2 Materials and methods

### 2.1 Study subjects

**Figure 1:**
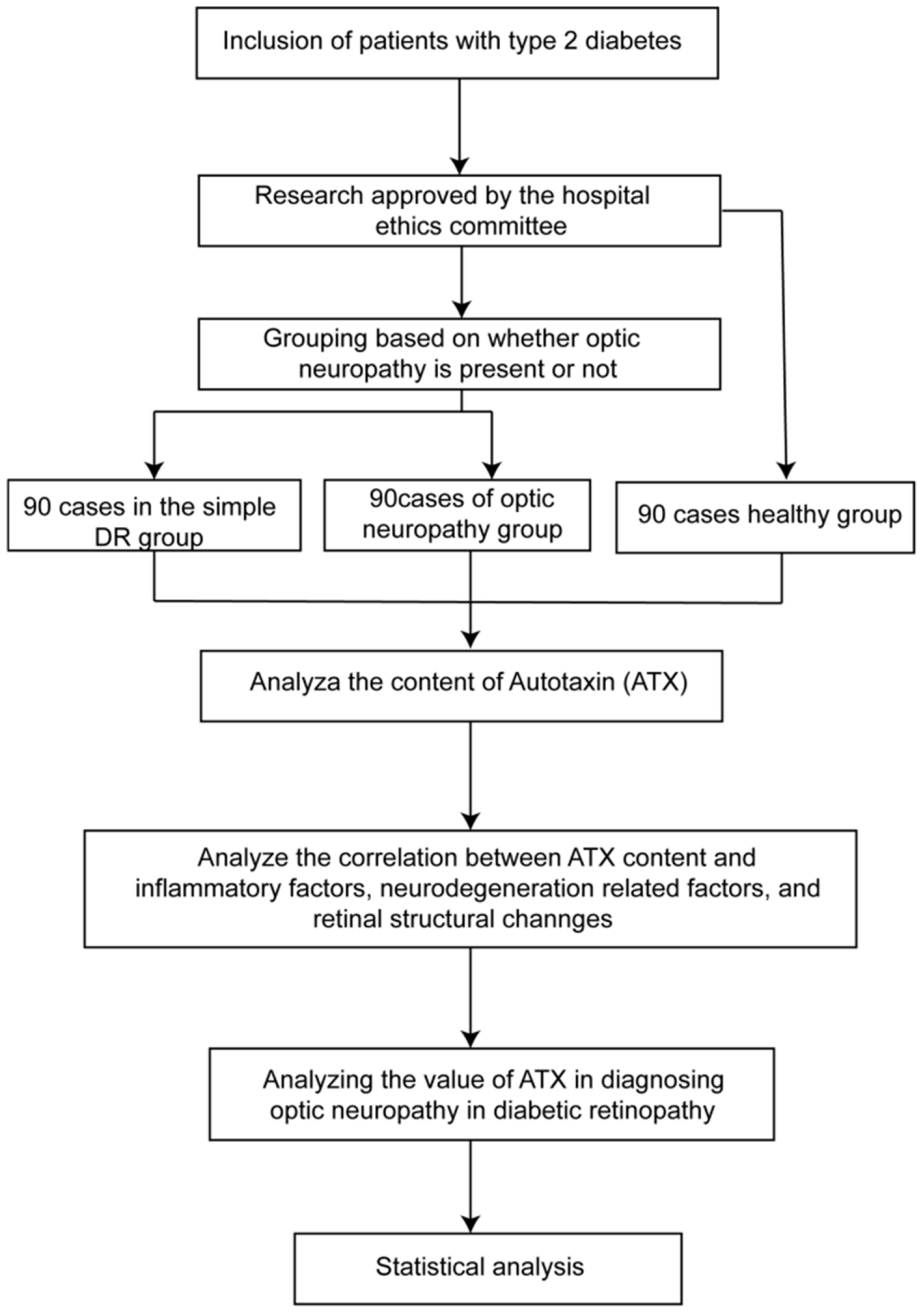
Study flow chart.

A total of 90 patients with early DR who were treated in our hospital from August 2022 to December 2023 were selected as the NDN group, and another 90 patients with early DR combined with optic neuropathy were selected as the combined DN group. In addition 90 healthy patients were selected as a normal control group. The study flow is shown in Figure 1. Inclusion criteria: (1) All patients met the diagnostic criteria for type 2 diabetes (9) and were diagnosed with early DR by fundus examination and optical coherence tomography (OCT) and other examinations; (2) Patients with combined optic neuropathy had ischemic optic neuropathy, optic disk neovascularization, optic nerve atrophy, and papilledema, and were diagnosed based on clinical symptoms, ophthalmological examination, slit lamp microscopy, dilated fundus examination, fluorescein fundus angiography, and multifocal electroretinogram (mf-ERG); (3) Age ≥ 18 years old, regardless of gender; (4) Complete clinical data and good compliance with examinations. Exclusion criteria: (1) Patients with other eye diseases such as cataract, glaucoma, blindness and conjunctivitis; (2) Patients with fundus diseases such as epiretinal membrane, retinal detachment, vitreous hemorrhage; (3) Patients with severe liver and kidney dysfunction; (4) Patients with malignant tumors or malnutrition; (5) Patients with severe acute or chronic complications of diabetes; (6) Patients with severe cardiovascular or cerebrovascular diseases such as coronary heart disease, stroke, aortic dissection, pulmonary embolism; (7) Patients with intraocular, intraorbital, intracranial, or systemic lesions that may cause optic disk edema. This study was approved by the Ethic Committee of Qingdao West Coast New District Central Hospital (IRB#0041-23-12R0).

### 2.2 Data collection

#### 2.2.1 Clinical data

The general data of the two groups of patients, including age, gender, body mass index (BMI), systolic blood pressure (SBP), diastolic blood pressure (DBP), and whether they had cataracts, were collected. Laboratory indicators included fasting blood glucose, white blood cell count (WBC), platelet count (PLT), total cholesterol (TC), triglycerides (TG), high-density lipoprotein cholesterol (HDL-C), low-density lipoprotein cholesterol (LDL-C), aspartate aminotransferase (AST), alanine aminotransferase (ALT), and serum creatinine (SCr) levels. The refraction of the two groups of patients was measured by an automatic refractor, the axial length was measured by an optical biometer, and the intraocular pressure level was measured by tonometer measurement.

#### 2.2.2 Inflammatory factors

The levels of various blood routine indicators of the two groups of patients were collected, and the platelet/lymphocyte ratio (PLR), neutrophil/lymphocyte ratio (NLR), lymphocyte/monocyte ratio (LMR), systemic inflammatory response index (SIRI), and platelet/albumin ratio (PAR) were calculated, where SIRI = (neutrophil/lymphocyte) × monocyte.

Enzyme-linked immunosorbent assay (ELISA) was used to detect serum levels of C-reactive protein (CRP), tumor necrosis factor-α (TNF-α), and interleukin-6 (IL-6) levels. The procedures were as follows: venous blood was drawn from the subjects, and the blood was naturally coagulated at room temperature for 10–20 min, then centrifuged at 2,000–3,000 rpm for about 20 min, and the supernatant was collected. EDTA or sodium citrate was used as an anticoagulant, after mixing, the sample was centrifuged again for 20 min, and the supernatant was collected. Standard solutions, test samples, and biotinylated detection antibodies were added to the wells of the pre-prepared ELISA plate, and streptavidin (Streptavidin-HRP) labeled with horseradish peroxidase was added. The samples were added to the pre-prepared wells, and the wells were washed with washing solution. A chromogen was added to each well to develop a blue color, followed by the addition of the substrate TMB, allowing for color development in the dark. The intensity of the color was proportional to the concentration of CRP, TNF-α, and IL-6 in the samples. A stop solution was added to change the color from blue to yellow, halting the reaction. The absorbance at 450 nm was read, and the concentrations of CRP, TNF-α, and IL-6 in the samples were calculated using a standard curve.

#### 2.2.3 ATX and neurodegeneration factors

Five milliliter of fasting venous blood was collected from the two groups of patients in the morning and centrifuged at 3,000 r/min for 5 min with a centrifugal radius of 10 cm. The supernatant was used to detect serum levels of ATX, glial fibrillary acidic protein (GFAP), and neurofilament light (NfL) using enzyme-linked immunosorbent assay (ELISA).

#### 2.2.4 Retinal OCT examination

Both groups of patients underwent retinal OCT examination, and the FAZ area of the subjects was recorded. The acircularity index (AI) was calculated (AI = the perimeter of the FAZ/the perimeter of a circle with equal area). The mean RNFL thickness at the disk margin, temporal, superior, nasal, and inferior were measured, with the mean of three measurements taken as the RNFL thickness for that eye. Additionally, the mean macular ganglion cell inner plexiform layer (mGCIPL) thickness and vascular density (VD) levels were recorded.

### 2.3 Statistical methods

Data were analyzed using SPSS 23.0 statistical software. Continuous variables were expressed as (x̄ ± s), and the comparison between the two groups was performed using the independent sample t test. Categorical variables were expressed as [cases (%)], and the chi-square test was used for comparisons between groups. The Pearson correlation test was used for correlation analysis. Multivariate logistic regression analysis was used to analyze the influencing factors of optic neuropathy in DR patients, and the ROC curve was used to analyze the clinical value of ATX in diagnosing optic neuropathy in DR patients. The statistical results were considered statistically significant when *p* < 0.05.

## 3 Results

### 3.1 Comparison of general data between the three groups of patients

There was no significant difference in general data between the patients with simple DR and those with combined optic neuropathy (*p* > 0.05), as shown in Table 1. The general data included age, gender, duration of diabetes, blood pressure, body mass index (BMI), blood lipid levels, etc. This finding suggests that in exploring the development mechanism of DR and its complications and formulating treatment strategies, it may be necessary to conduct a more in-depth investigation of other non-general biological markers or genetic factors in order to more accurately understand the disease process and individual differences.

**Table 1:** Comparison of general data between the three groups (x̄ ± s, %).

General data		Healthy group (*n* = 90)	NDN group (*n* = 90)	DN group (*n* = 90)	*t*	*P*
Gender	Male	55 (61.11)	56 (62.22)	50 (55.56)	0.413	0.520
	Female	35 (38.89)	34 (37.78)	40 (44.44)		
Age (years)		57.99 ± 8.70	59.04 ± 9.16	58.71 ± 9.00	0.345	0.806
BMI (kg/m^2^)		26.71 ± 2.93	25.74 ± 2.23	25.62 ± 2.31	0.725	0.796
Diabetes duration (years)		6.80 ± 2.19	5.18 ± 2.23	6.01 ± 2.31	1.522	0.075
SBP (mmHg)		121.51 ± 11.48	120.751 ± 12.22	120.97 ± 11.92	0.555	0.895
DBP (mmHg)		72.30 ± 6.02	73.30 ± 6.18	72.76 ± 6.18	0.951	0.564
Diopter (D)		0.63 ± 0.60	0.68 ± 0.54	0.68 ± 0.48	0.654	0.976
Axis length (mm)		22.06 ± 0.88	21.98 ± 0.86	21.97 ± 0.86	1.362	0.836
Intraocular pressure (mmHg)		15.91 ± 4.42	16.18 ± 4.76	15.68 ± 4.41	1.541	0.462
Cataract	Combined	40 (44.44)	30 (33.33)	42 (46.67)	1.667	0.198
	Not combined	50 (55.56)	60 (66.67)	48 (53.33)		
Fasting blood glucose (mmol/L)		8.11 ± 0.65	7.94 ± 0.68	8.10 ± 0.65	1.396	0.116
WBC (×10^9^/L)		6.53 ± 0.74	6.41 ± 0.80	6.52 ± 0.85	1.210	0.346
PLT (×10^9^/L)		236.914 ± 22.31	235.44 ± 21.36	237.01 ± 24.63	0.534	0.658
TC (μmol/L)		1.53 ± 0.29	1.51 ± 0.28	1.51 ± 0.29	0.531	0.922
TG (μmol/L)		4.70 ± 0.44	4.69 ± 0.51	4.69 ± 0.47	0.452	0.996
HDL-C (μmol/L)		1.39 ± 0.20	1.41 ± 0.18	1.37 ± 0.20	0.309	0.183
LDL-C (μmol/L)		3.22 ± 0.49	3.22 ± 0.37	3.18 ± 0.41	0.329	0.466
AST (U/L)		22.89 ± 3.33	23.05 ± 3.69	23.34 ± 3.42	1.624	0.583
ALT (U/L)		21.00 ± 3.17	21.28 ± 2.86	20.60 ± 2.86	1.430	0.113
SCr (μmol/L)		58.11 ± 11.31	568.50 ± 11.21	56.27 ± 9.71	0.756	0.149

### 3.2 Comparison of systemic inflammatory index and inflammatory factors between the three groups

There was no statistically significant difference in the levels of PLR, NLR, LMR, PAR, SIRI index and serum inflammatory and metabolic indicators such as TNF-α, CRP, and IL-6 between the patients in the NDN group and the patients in the combined DN group (*p* > 0.05), as shown in Table 2. These results show that within the sample range of the current study, the NDN group and the combined DN group did not show differences in the above physiological and biochemical indicators that could clearly distinguish the two groups, suggesting that further exploration of other more sensitive biological markers or indicators may be needed to better understand the pathogenesis and individual differences of DR and its complications.

**Table 2:** Comparison of systemic inflammatory index and inflammatory factors between the three groups (x̄ ± s).

Inflammatory markers	Healthy group (*n* = 90)	NDN group (*n* = 90)	DN group (*n* = 90)	*t*	*P*
PLR	137.15 ± 16.53	139.48 ± 16.70	133.87 ± 17.61	0.465	0.530
NLR	3.16 ± 0.55	2.98 ± 0.58	3.07 ± 0.46	0.785	0.240
LMR	3.13 ± 0.53	3.07 ± 0.64	3.06 ± 0.60	0.974	0.892
PAR	4.68 ± 1.52	4.53 ± 1.58	4.52 ± 1.52	0.196	0.951
SIRI	0.88 ± 0.16	0.78 ± 0.15	0.81 ± 0.17	1.450	0.159
TNF-α (ng/L)	119.99 ± 17.54	119.15 ± 13.58	119.46 ± 16.24	0.562	0.920
CRP (mg/L)	5.72 ± 1.28	5.73 ± 1.25	5.80 ± 1.20	1.005	0.712
IL-6 (pg/mL)	15.38 ± 3.61	14.62 ± 3.70	15.52 ± 3.51	1.239	0.096

### 3.3 Comparison of ATX and neurodegeneration-related factors between the three groups

**Figure 2:**
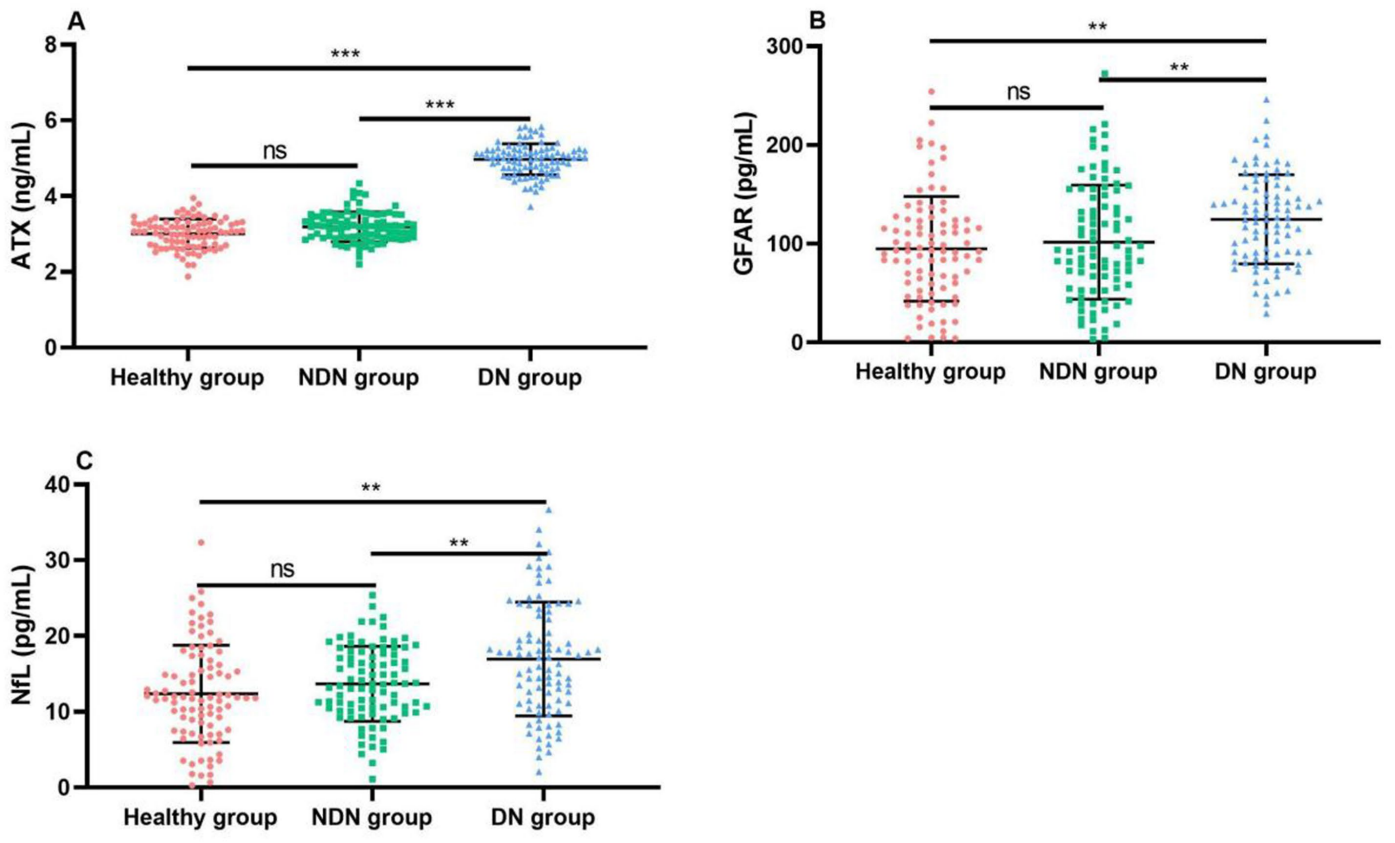
Comparison of ATX and neurodegeneration-related factor levels between the three groups of patients. **(A)** Comparison of ATX levels between the two groups; **(B)** Comparison of GFAP levels between the two groups; **(C)** Comparison of NfL levels between the two groups. Compared with the NDN group *p < 0.05, **p < 0.01, ***p < 0.001.

NfL levels of patients with combined optic neuropathy were significantly higher than those of patients with simple DR, and the difference was statistically significant (*p* < 0.05). This finding suggests that the three biomarkers ATX, GFAP, and NfL may play an important role in the pathogenesis of DR, and their increase may be related to optic nerve damage, inflammatory response, or glial cell activation. Therefore, the detection of these indicators may help us identify high-risk patients with combined optic neuropathy earlier, so as to take more targeted intervention measures to delay disease progression and improve patients’ quality of life, as shown in Table 3 and Figure 2.

**Table 3:** Comparison of ATX and neurodegeneration-related factors between the three groups of patients (x̄ ± s).

Factor	Healthy group (*n* = 90)	NDN group (*n* = 90)	DN group (*n* = 90)	*t*	*P*
ATX (ng/mL)	3.03 ± 0.39	3.19 ± 0.39	4.97 ± 0.41	13.519	<0.001
GFAP (pg/mL)	99.63 ± 53.58	101.64 ± 57.70	124.75 ± 45.20	1.693	0.003
NfL (pg/mL)	12.88 ± 6.39	13.70 ± 4.96	16.96 ± 7.53	2.343	0.001

### 3.4 Comparison of retinal OCT examination results between the two groups of patients

The mGCIPL and VD levels of patients with combined optic neuropathy were significantly lower than those of patients with simple DR, and this difference was statistically significant (*p* < 0.001). This finding indicates that in diabetic patients, when combined with optic neuropathy, the ganglion cell layer in the macular area may have suffered more severe damage, and the vascular structure of the retina may have also changed significantly, resulting in a decrease in vascular density. As important indicators for evaluating retinal structure and function, mGCIPL and VD, their significant decrease may indicate an aggravation of visual impairment, as shown in Table 4.

**Table 4:** Comparison of retinal OCT examination results between NDN group and DN group (x̄ ± s).

Factor	NDN group (*n* = 90)	DN group (*n* = 90)	*t*	*P*
Temporal quadrant RNFL (μm)	85.23 ± 6.33	85.74 ± 6.04	0.631	0.582
Superior quadrant RNFL (μm)	124.48 ± 12.05	122.08 ± 14.90	0.865	0.236
Nasal quadrant RNFL (μm)	83.21 ± 11.63	84.66 ± 11.73	0.652	0.405
Inferior quadrant RNFL (μm)	120.41 ± 11.00	117.49 ± 11.45	0.845	0.084
Mean RNFL thickness at the disk margin (μm)	105.62 ± 7.36	105.85 ± 6.57	1.550	0.819
FAZ area (mm^2^)	0.49 ± 0.11	0.46 ± 0.12	0.389	0.172
AI	0.58 ± 0.09	0.57 ± 0.10	0.268	0.976
mGCIPL (μm)	87.29 ± 4.44	81.80 ± 6.19	5.361	<0.001
VD (%)	47.52 ± 2.04	44.54 ± 2.113	3.597	<0.001

### 3.5 Correlation analysis between ATX levels and neural factors in DR patients

**Figure 3:**
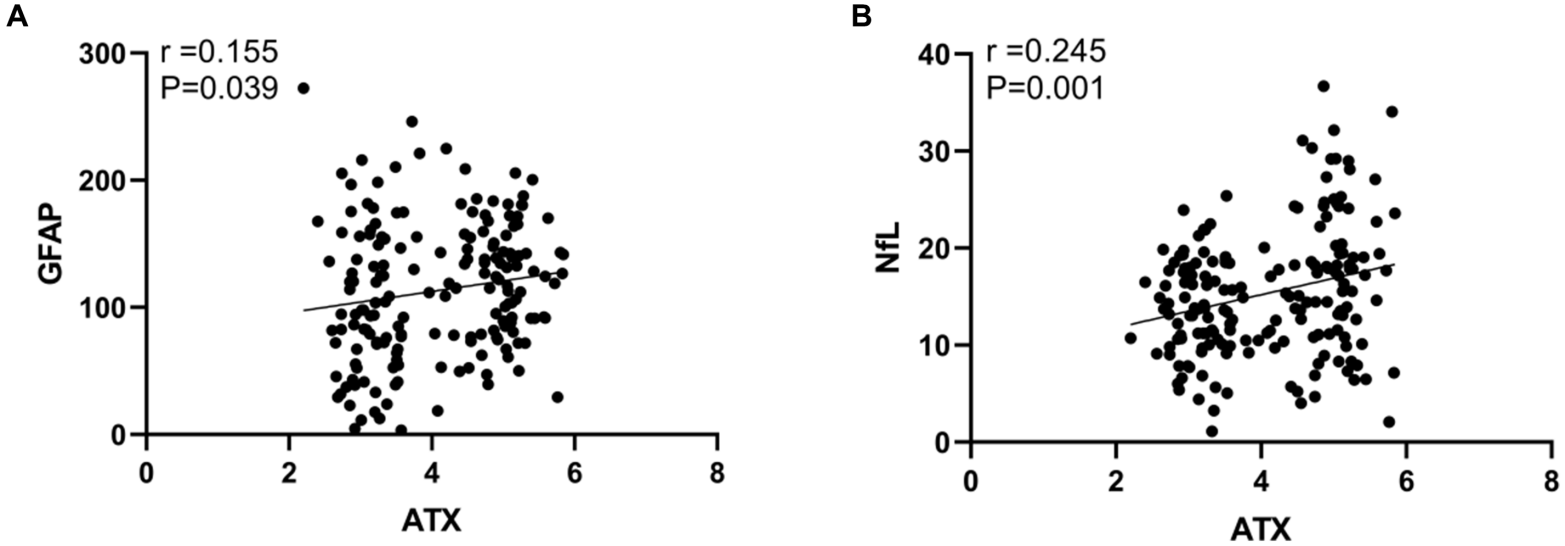
Correlation analysis between ATX levels and neural factors in DR patients. **(A)** Correlation between ATX level and GFAP level; **(B)** Correlation between ATX level and NfL level.

The results of Pearson correlation analysis showed that the ATX level in DR patients was positively correlated with GFAP and NfL. Specifically, the correlation coefficient between ATX and GFAP was 0.155, and the correlation coefficient with NfL was 0.245, and both correlations were statistically significant (*p* < 0.05). As the ATX level increased, the levels of GFAP and NfL also tended to increase, which may suggest that ATX, GFAP, and NfL may have synergistic effects or mutual correlations in the pathophysiological process of DR (see Figure 3).

### 3.6 Correlation between ATX levels and retinal OCT examination results in DR patients

**Figure 4:**
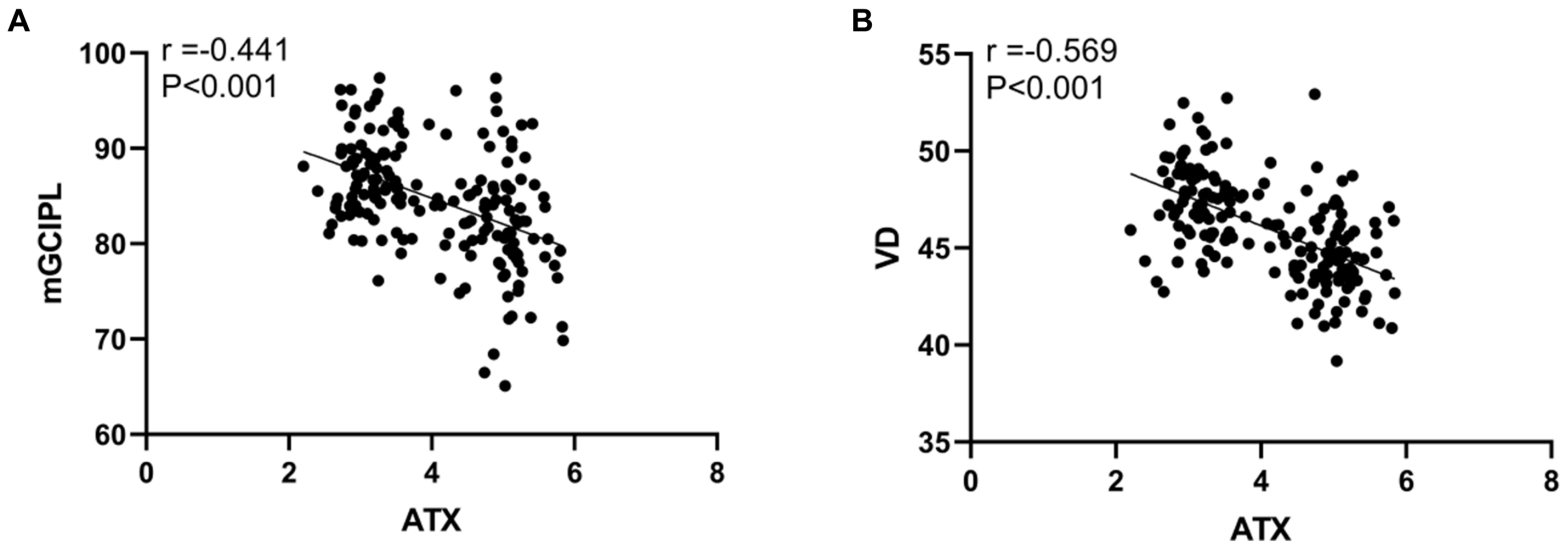
Correlation between ATX levels and retinal OCT examination results in DR patients. **(A)** Correlation between ATX level and mGCIPL level; **(B)** Correlation between ATX level and VD level.

The results of Pearson correlation analysis showed that the ATX level in DR patients showed a significant negative correlation with the mGCIPL and VD levels. Specifically, the correlation coefficient between ATX and mGCIPL was −0.441, while the correlation coefficient with VD reached −0.569, and both negative correlations were statistically highly significant (*p* < 0.001). This shows that as the ATX level increases, the mGCIPL and VD levels of DR patients tend to decrease, which may mean that the increase in ATX is closely related to the damage to the retinal structure and function (see Figure 4).

### 3.7 Multivariate logistic regression analysis of influencing factors of optic neuropathy in DR patients

Multivariate Logistic regression analysis was performed with whether DR patients developed optic neuropathy as the dependent variable and the statistically significant indicators in Tables 1–4 as independent variables. The results showed that serum ATX, GFAP, and NfL levels were independent risk factors for optic neuropathy in DR patients (*p* < 0.05), and GFAP and NfL were independent protective factors (*p* < 0.05), as shown in Table 5.

**Table 5:** Multivariate logistic regression analysis of influencing factors of optic neuropathy in DR patients.

Factors	*B* value	Standard error	*Wald* value	*P*-value	Odds ratio	95% *CI*
ATX	2.124	0.459	7.457	<0.001	9.751	3.509 ~ 26.473
GFAP	1.984	0.782	6.062	<0.001	4.340	1.640 ~ 17.286
NfL	2.569	0.129	8.365	<0.001	7.695	6.896 ~ 11.970
mGCIPL	−0.359	0.131	12.687	<0.001	0.883	0.640 ~ 0.957
VD	−1.829	0.684	7.127	0.006	0.462	0.238 ~ 0.683

### 3.8 ROC curve analysis of the clinical value of ATX in diagnosing optic neuropathy in DR patients

**Figure 5:**
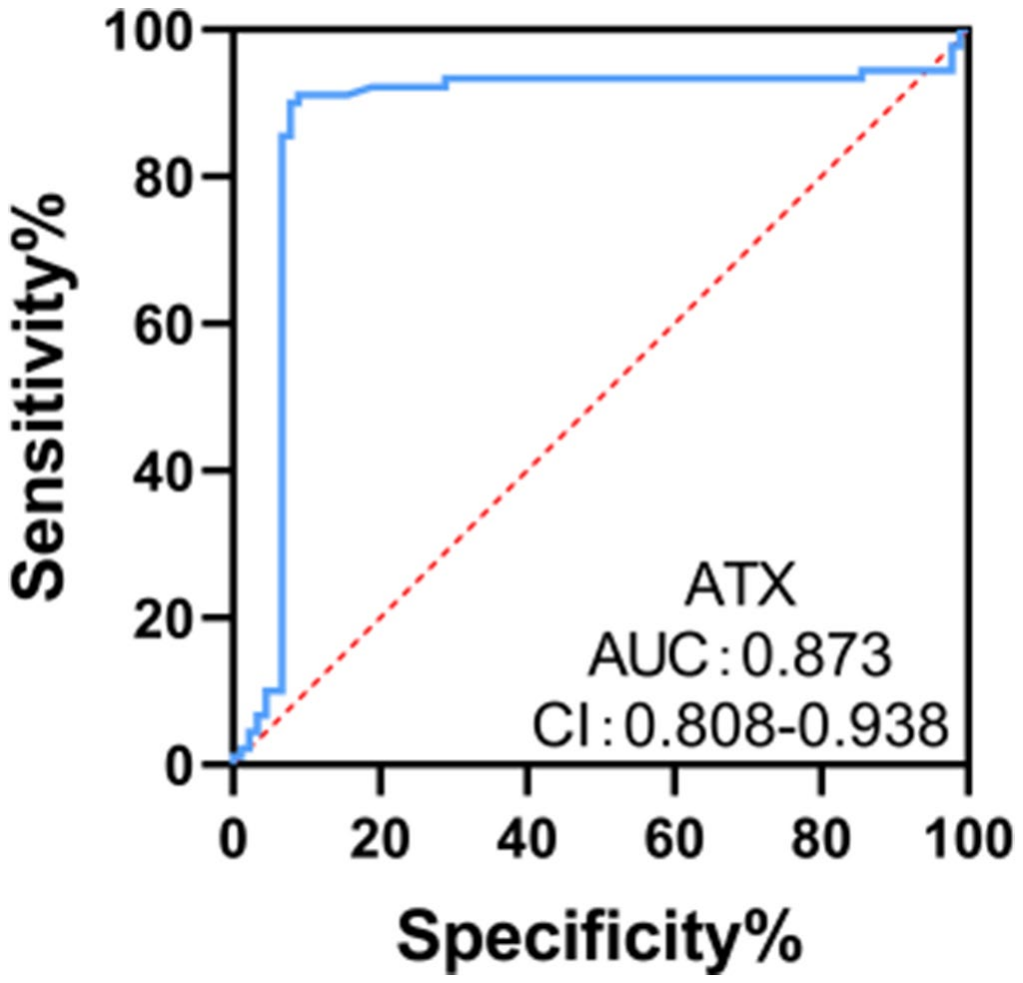
ROC curve analysis of the clinical value of ATX in diagnosing optic neuropathy in DR patients.

The ROC curve analysis results showed that the area under the curve (AUC) of ATX for diagnosing optic neuropathy in DR patients was 0.873, with a 95% CI of 0.808–0.938. When the ATX cut-off value was 4.69 ng /mL, the Youden index was 0.635 at the maximum, with a sensitivity of 71.11% and a specificity of 93.33%. It has a certain clinical value in diagnosing optic neuropathy in DR patients (see Figure 5).

## 4 Discussion

DR is caused by damage to the retinal microvasculature due to hyperglycemia, which leads to vascular leakage and blockage, thus causing fundus lesions. This lesion usually has no obvious symptoms in the early stages, but as the disease progresses, it may cause serious vision problems or even blindness. The manifestations of early diabetic optic neuropathy include optic disk neovascularization and ischemic optic neuropathy, and patients may feel that their vision is declining (10). In the early stages of DR, although the retinal microvessels are mainly affected, the optic nerve may also be indirectly affected. The hyperglycemia state caused by diabetes can damage the retinal microvessels, causing pathological changes such as thickening of the vascular wall, stenosis of the lumen, and slowed blood flow. These changes may cause retinal ischemia and hypoxia, and then produce neovascularization. The formation of neovascularization not only increases the risk of retinal hemorrhage and exudation, but may also cause compression or damage to the optic nerve (11, 12). Early diagnosis and treatment of optic neuropathy in DR is crucial to protecting patients’ vision.

Hyperglycemia in diabetic patients activates a variety of inflammatory factors, such as leukocyte adhesion molecules, inflammatory mediators, and micromicelle proteins. These inflammatory factors induce microglia to release proinflammatory cytokines, such as TNF-α, through different signaling pathways, such as the NF-κB and ERK pathways, thereby exacerbating the inflammatory response (13). In addition, the levels of proinflammatory factors such as IL-1β, IL-6, and IL-8 increase under high glucose conditions, further triggering the activation of microglia and leading to apoptosis of retinal ganglion cells (14). Under high glucose conditions, oxidative stress increases significantly, leading to increased intracellular NADPH oxidase activity, decreased SOD activity, decreased GSH levels, and increased lipid peroxide accumulation. Oxidative stress not only directly damages retinal neurons, but also activates inflammatory signaling pathways to promote the release of inflammatory factors, further exacerbating the inflammatory response (15). The results of this study showed that there were no statistically significant differences in PLR, NLR, LMR, PAR, SIRI indexes and serum TNF-α, CRP, and IL-6 levels between the NDN group and the combined DN group. Inflammatory indicators are indeed involved in the process of optic neuropathy in DR patients, but these indicators did not show sufficient specificity and could not be used as specific diagnostic indicators.

ATX is a secretory lysophospholipase D, whose main function is to catalyze the hydrolysis of lysophosphatidylcholine (LPC) to LPA. LPA is a water-soluble phospholipid similar to a growth factor, which has multiple cell activities such as cell proliferation, survival, migration, cytokine and chemokine production, and smooth muscle cell transformation. As a signaling factor, it is related to metabolic and inflammatory diseases (16). LPA plays a particularly significant role in the occurrence and development of diabetes and its complications (17). Studies have shown (18) that diabetic nephropathy (DN) is one of the most common microvascular complications of diabetes, and LPA signaling plays a key role in renal fibrosis, thereby inducing diabetic nephropathy. Abu El-Asrar et al. (19) found that ATX and LPA accelerate the movement of endothelial cells and promote the regression of new blood vessels during the proliferative stage of diabetic retinopathy. Therefore, as a marker of DR, a decrease in their content indicates an increase in new blood vessels and the entry of DR into the proliferative stage. Liu et al. (20) found that ATX is highly expressed in the retinal pigment epithelium and regulates the function of photoreceptor cells by secreting LPA to maintain the stability of the retinal interlayer structure and function. A study found (21) that the ATX/LPA pathway affects the prognosis of glaucoma filtering surgery due to its fibrotic effect. The role of the ATX/LPA pathway may be similar to the pathological cause of open-angle glaucoma, namely trabecular meshwork cell fibrosis and cytoskeletal changes. The results of this study showed that the serum ATX, GFAP, and NfL levels of patients with combined optic neuropathy were significantly higher than those of patients with simple DR, and ATX was positively correlated with GFAP and NfL levels, indicating that serum ATX content was closely related to optic neuropathy in early DR. The reason for this may be that ATX and LPA play an important role in angiogenesis, mitosis, and cell proliferation. It is believed that ATX may aggravate the progression of diabetic retinopathy by promoting abnormal proliferation and leakage of retinal blood vessels. In addition, in the diabetic state, ATX expression increases, leading to increased LPA levels. LPA acts on insulin receptors or their downstream signaling pathways, inhibiting insulin sensitivity, thereby aggravating insulin resistance, which may lead to reduced utilization of glucose by retinal tissue and aggravate retinal ischemia and hypoxia (22, 23).

Studies have shown (24) that in DR patients, as the disease progresses, the thickness of the mGCIPL gradually becomes thinner. This change may be related to the damage and apoptosis of retinal ganglion cells. When the thickness of the mGCIPL decreases, the processing and transmission of visual information may be disturbed, leading to a decline in optic nerve function. In addition, some studies have also found (25) that after treatment, although the central macular thickness (CMT) of diabetic macular edema (DME) patients has good anatomical reposition, the thickness of the mGCIPL is still lower than that of DR patients without DME, and is related to vision, which further confirms the importance of mGCIPL thickness in DR optic neuropathy. Studies have shown (26) that in DR patients, as the disease progresses, the retinal vascular density gradually decreases. This change may be related to the damage and occlusion of retinal blood vessels. When the vascular density decreases, the blood perfusion of the retina may be affected, resulting in retinal ischemia and hypoxia, which in turn aggravates the damage and pathology of the optic nerve. The results of this study showed that the mGCIPL and VD levels of patients with combined optic neuropathy were significantly lower than those of patients with simple DR, and ATX was negatively correlated with mGCIPL and VD levels, that is, with the increase of ATX levels, the thickness of mGCIPL will gradually decrease, and the vascular density will also decrease, which may be related to ATX promoting retinal inflammatory response, oxidative stress and other pathological processes. These pathological processes will aggravate retinal neurodegeneration and vascular lesions, thereby leading to a decrease in mGCIPL thickness and vascular density.

ATX is highly expressed in a variety of tumors and promotes tumor cell growth and migration by converting LPC to LPA. In the multiforme glioblastoma cell line U87-MG, ATX supports tumor cell proliferation and invasion by promoting LPA production (27). In addition, in pancreatic cancer patients, the expression levels of serum ATX and LPA are clinically significant for the diagnosis of resectable and borderline resectable pancreatic cancer (28). ATX is closely related to liver fibrosis. Studies have shown that serum ATX levels are significantly increased in patients with chronic hepatitis B and are positively correlated with the degree of liver fibrosis, showing a high diagnostic value (29). Serum ATX and LPA levels are significantly increased in patients with hepatocellular carcinoma and are related to the degree of differentiation, indicating that ATX may be involved in the development of liver fibrosis and hepatocellular carcinoma, and may serve as an independent risk factor for recurrence after surgical treatment (30). The results of ROC curve analysis in this study showed that the area under the curve (AUC) of ATX for diagnosing optic neuropathy in DR patients was 0.867, indicating that serum ATX levels have certain clinical value in diagnosing optic neuropathy in DR patients and can be used as an independent factor and potential marker for neuropathy, providing potential for disease management. ATX participates in signal transduction through LPA receptors, which can induce cell proliferation, migration, secretion of cytokines and chemokines, and reduction of cell apoptosis, making it play an important role in a variety of physiological and pathological processes (31). Related data show (32) that ATX may also directly damage optic nerve cells, leading to abnormalities in their structure and function. Optic nerve cells are important cells in the retina responsible for transmitting visual information. The integrity of their structure and function is crucial to maintaining normal visual function. Overexpression of ATX may directly damage their structure and function by affecting the metabolism and signal transduction pathways of optic nerve cells.

ATX and lysophosphatidic acid (LPA) play important roles in angiogenesis, mitosis and cell proliferation. ATX may exacerbate the progression of diabetic retinopathy by promoting abnormal proliferation and leakage of retinal vessels. In the diabetic state, ATX expression is increased, leading to elevated levels of LPA. LPA acts on insulin receptors or their downstream signaling pathways to inhibit insulin action, thereby exacerbating insulin resistance and metabolic abnormalities. These abnormalities may lead to retinal ischemia and hypoxia, which in turn exacerbate optic nerve damage and pathological changes. There is a correlation between ATX and optic nerve damage, but more scientific evidence and in-depth studies are needed to directly establish a causal relationship.

In summary, increased serum ATX levels may be involved in the occurrence and development of optic neuropathy in DR, and have a significant correlation with nerves and blood vessels in the early stages of the disease. Detection of serum ATX levels is helpful for the early diagnosis of optic neuropathy in DR and provides new ideas for its treatment.

This study has certain limitations. The sample size of this study may not be sufficient to represent the entire population of patients with early diabetic retinopathy, especially when the study involves specific subgroups (such as different ages, genders, disease duration, etc.). In addition, there may be bias in sample selection, which may lead to the results not being universal. The study did not include normal controls, and the follow-up analysis results were missing. The study did not consider all confounding factors that may affect serum Autotaxin levels and optic neuropathy, such as genetic background, lifestyle, and other complications. Therefore, it is necessary to further expand the sample size in the future and conduct multicenter, prospective studies to verify the correlation between serum ATX levels and optic neuropathy in early diabetic retinopathy and explore its application value in clinical practice.

## Data Availability

The original contributions presented in the study are included in the article/supplementary material, further inquiries can be directed to the corresponding author.
